# Exploring the Benefit and Sacrifice Factors of Virtual Reality Gameplay

**DOI:** 10.3389/fpsyg.2020.00251

**Published:** 2020-03-10

**Authors:** Chun-Chia Lee, Kuo-Lun Hsiao, Chia-Chen Chen

**Affiliations:** ^1^School of Business, Minnan Normal University, Zhangzhou, China; ^2^Department of Information Management, National Taichung University of Science and Technology, Taichung, Taiwan; ^3^Department of Management Information Systems, National Chung Hsing University, Taichung, Taiwan

**Keywords:** benefit value, gameplay intention, flow, sacrifice factor, virtual reality

## Abstract

Although Virtual reality (VR) entertainment is now relatively popular, the adoption of VR devices is still low. In this study, a framework based benefit and sacrifice factors was developed to understand players’ intention to use VR devices to play games. Online questionnaire items were developed and published to collect the responses from university students in Taiwan. The feedback of 152 inexperienced players and 150 experienced players were collected. The eleven hypotheses were tested by using SmartPLS, a structural equation modeling (SEM) tool. The analysis results show that the influences of the benefit factors (flow, spatial presence, and relaxation) on the adoption intention in two groups are consistent. All of the positive influences are supported. Moreover, visual fatigue had the strongest negative effects on flow and intention. Players who are opener to new technology are more possible to adopt VR devices. The findings can provide insights to VR device developers to design their VR devices/contents and marketing strategies.

## Introduction

Virtual reality (VR) is an interactive, simulated visual and audio environment generated by computer technology. This environment can be a fantastic world or one that resembles the real world. In the immersive environment, users can interact and play with virtual objects, either alone or with other users. VR techniques have been adopted in fields such as e-learning, healthcare, automotive applications, and gaming. According to SuperData Research Holdings, Inc. (SuperData, 2019), revenue for VR software and hardware will reach $37 billion (USD) by 2020. The total earnings of VR have risen significantly in recent years.

The most common VR applications use a VR headset to display a realistic visual environment. The headset incorporates sound and various sensors to simulate the user’s physical presence in the computer-generated environment. The types of the VR headsets are console-tethered and mobile devices. Console-tethered headsets usually have better performance and provide a more immersive experience, while mobile headsets are relatively inexpensive and easier to use. A console-tethered headset must be connected directly to a personal computer or game console, whereas a mobile VR headset can be used wirelessly with a smartphone. To entice more users to adopt VR headsets, companies such as Facebook, Google, and hTC are developing cheaper mobile VR devices. Though mobile VR headsets are more convenient, their low resolution and refresh rates can cause visual fatigue. VR content has certain advantages, such as the ability to create interest and increase engagement. However, the use of VR can also cause simulator sickness or addiction. Hence, understanding what factors drive consumers to adopt a VR headset to watch/play VR content is very important.

Virtual reality entertainment has grown in popularity, and VR tools have become increasingly available and accessible (Alcañiz et al., 2019). The virtual environment generated by VR can help users relax their body and/or mind. Van Kerrebroeck et al. (2017) pointed out that shopping in a virtual environment can decrease customers’ perceptions of crowding, in contrast to their experience in shopping malls. Higher levels of perceived crowding have been shown to decrease user satisfaction and loyalty. Didehbani et al. (2016) also found that user interactions in VR can train and enhance users’ social abilities. Serrano et al. (2013) demonstrated that virtual environments can significantly help users relax their body and mind, and found that user satisfaction with such technology is high. However, the overuse of VR technology can cause VR sickness. Some users experience headaches, nausea, or virtual fatigue. Such negative effects can decrease users’ intention to adopt VR technology.

Value-related theories have been adopted in many consumer intention studies to explore the determinants of shopping or adoption intention. Value, in this context, is defined as the result of evaluating the benefits and sacrifices generated by a behavior. For example, Hsiao and Chen (2018) explored how social, quality, emotional and price values affect smartphone adoption intention. These same values are also the key drivers of the payment intention in mobile app services (Hsiao and Chen, 2016). Value-related factors are also important determinants of the usage of social networking and e-book subscription services (Hsiao and Chen, 2017). Moreover, the explanatory power of these values on behavioral intention is relatively high (Hsiao and Chen, 2018). Thus, perceived value theory is adopted as the base model of the research framework in this study.

In the VR context, the main benefits which users can experience are flow, spatial presence, and relaxation. Past research has shown that possible sacrifices include visual fatigue and the complexity of the VR technology. To gain further insight into users’ intention to adopt VR products needs further investigation into the potential relationships between the drivers. VR games are the most popular VR applications. Therefore, this study will explore these factors in the context of VR games. In summary, the research purpose in this study is twofold. First, we investigate the benefit/sacrifice factors that contribute to the adoption of VR headsets for gameplay. Second, we explore the factor differences between experienced players and inexperienced players. This investigation can help VR product manufacturers understand how to motivate consumers to adopt their products.

## Research Model and Hypotheses

**FIGURE 1:**
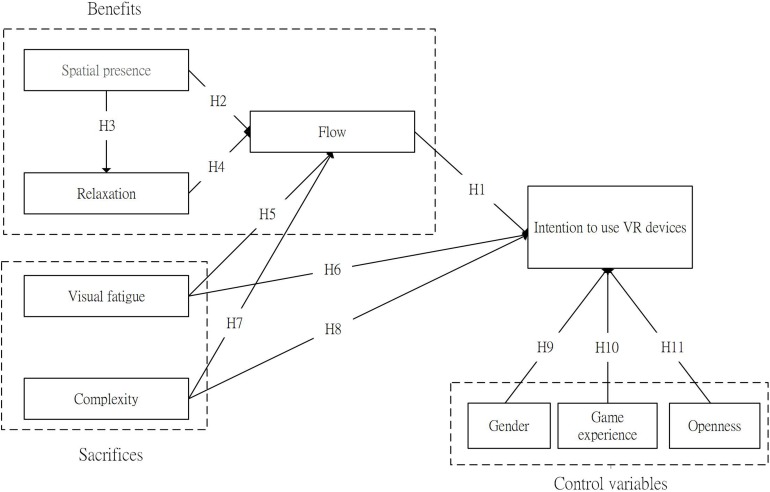
Research model.

We propose a VR device adoption framework based on perceived value theory as follows. The model includes both benefit- and sacrifice-related factors (see Figure 1). The benefit factors are spatial presence, relaxation, and flow. The spatial presence is the most critical and beneficial attribute of VR technology, which will enhance the other two key factors, flow and relaxation, in a game (Lee et al., 2018). The sacrifice factors are visual fatigue and complexity, which are the two drawbacks usually mentioned. The control variables for adoption intention are gender, game experience, and openness.

### Flow

Flow is a state of optimal pleasure that occurs when people concentrate on an activity. The flow construct in the model is measured using items proposed by Koufaris (2002). The perceived enjoyment and concentration-related items were adopted to evaluate users’ optimal pleasure state. Perceived enjoyment is the extent to which an individual perceives using VR devices to play games as enjoyable (Lu et al., 2009; Zhou and Lu, 2011). Past studies have demonstrated that users’ perceived enjoyment has a strong effect on technology adoption, especially in computer game and leisure contexts. In this study, concentration refers to the extent to which an individual is able to focus on using VR devices to play games (Lu et al., 2009; Zhou and Lu, 2011). When people are more focused on playing in a VR context, they will find it easier to experience a state of optimal pleasure. In the VR gameplay context, the experience will bring more pleasure and enhance users’ intention to use VR devices. Therefore, hypothesis 1 is proposed.

H1: Flow will positively affect the players’ intention to adopt VR devices to play games.

### Spatial Presence

Users’ perception of spatial presence refers to the experience of believing they have been transported elsewhere (Rodríguez-Ardura and Meseguer-Artola, 2016). When people use VR devices, they feel like they have been transported to the VR environment. The influence of spatial presence has been widely discussed in different information system usage contexts. Franceschi et al. (2009) demonstrated that spatial presence significantly affects flow. Rodríguez-Ardura and Meseguer-Artola (2016) also proved that users’ perception of spatial presence had strong effects on both flow and online learning usage intention. In the context of VR, spatial presence can help the user to relax. Many companies have developed relaxing VR games in order to entice players to adopt VR devices. Some of the most popular relaxation VR games in 2018 were Chroma Lab, Tilt Brush, and Luna (PC Gamer, 2018). The popularity of such VR games indicates that spatial presence in a comfortable virtual environment can help users relax. Accordingly, hypothesis 2 and 3 are proposed.

H2: Spatial presence will positively affect the players’ flow perception.

H3: Spatial presence will positively affect the players’ relaxation.

### Relaxation

Relaxation is the extent to which users perceive themselves as experiencing a state of low tension. Relaxation is an important motivation for leisure activities, such as watch online video, playing computer games, and listening to music (Park et al., 2011; Wen et al., 2018). Relaxation can help individuals to be more involved in an activity. Moreover, a relaxed state can enhance the player’s ability to experience more pleasure in a game. Past research also demonstrated that users can feel relaxed in VR and have pleasure experience (Pizzoli et al., 2019). Thus, hypothesis 4 is proposed.

H4: Relaxation will positively affect the players’ flow perception.

### Visual Fatigue

One way to induce visual fatigue is to force the eye to focus on a small region for a prolonged period of time. Excessive time spent watching screens is a typical cause of visual fatigue. Past research has found that watching 3D videos and digital reading can lead to visual fatigue (Benedetto et al., 2014). Recently, the popularity of online videos and mobile games has increased the amount of time people spend staring at a smartphone screen. Therefore, the negative effects of visual fatigue on health have become a more significant issue. Watching the screen of a VR head-mounted display is likely to create similar problems, such as pain in the neck/shoulders, blurred vision, and difficulty focusing. Given these risks, users might hesitate to use the devices to play VR games. Moreover, visual fatigue can also lead to lack of concentration or reduce the user’s involvement in an activity. Therefore, hypothesis 5 and 6 are proposed.

H5: Visual fatigue will positively affect the players’ flow perception.

H6: Visual fatigue will negatively affect the intention to use VR devices to play games.

### Complexity

Complexity is the user’s perception of the level of difficulty involved in operating the VR device. Olatokun and Igbinedion (2009) found that complexity negatively affects users’ attitude toward technology as well as their usage intention. Complexity is one of the main factors users consider before adopting a new product/service (Chung et al., 2015). Though applications of VR have continued to expand in recent years, many people are still not familiar with VR products. Therefore, people will evaluate the complexity of using such a system before deciding whether or not to adopt this technology. A high level of complexity can also reduce the user’s pleasure and involvement in using the technology. Thus, hypothesis 7 and 8 are listed as below.

H7: Complexity will negatively influence the players’ flow perception.

H8: Complexity will negatively influence the intention to use VR devices to play games.

### Control Variables

In this research context, we consider gender, game experience, and openness as control variables which might influence usage intention. In a digital game context, usage and payment intention may be different among male and female players. For example, Hsiao and Chen (2016) found that female players in a paying-player group demonstrate a greater intention to make in-app purchases. For most digital games, male users outnumber female users. Furthermore, players whose personalities make them more open to new experiences are more likely to adopt new technology. Experienced players are also more likely to play VR games. Therefore, hypothesis 9, 10, and 11 are listed as below.

H9: Female players have a less intention to adopt VR devices to play games.

H10: Experienced players are more possible to adopt VR devices to play games.

H11: The personality trait of openness will positively affect the intention to adopt VR devices to play games.

## Research Method

Since most VR devices and applications are not popular, this study collected two different samples to understand the differences among different players. The first sample was comprised of less experienced VR game players. This sample was collected from a university in central Taiwan. The student players were invited to use VR devices to play a VR game in a meeting room. The VR devices used were Oculus Rift and Samsung Gear VR. The VR games played were interactive music games. Before game play, a research assistant taught the participants how to use the devices to play the game. Once participants had played the games, they filled out an online questionnaire, which took approximately 25 min. We collected a total of 152 responses for this sample. The second sample was comprised of experienced VR game players. This sample was collected from popular online VR communities in Taiwan, such as VRChat and hTC VIVEPORT. Hyperlinks to the online questionnaire were posted on these communities to invite experienced players to answer the questions in the questionnaire. A total of 150 valid surveys remained after deleting duplicates and incomplete responses.

A five-point Likert scale was used for all items, ranging from “strongly disagree” (1) to “strongly agree” (5). The items for the flow were adapted from Lu et al. (2009). Items for measuring relaxation were adapted from Kozak (2002) and Wen et al. (2018). Spatial presence was assessed based on the scale proposed by Rodríguez-Ardura and Meseguer-Artola (2016). Items for measuring visual fatigue were taken from Cho et al. (2014). The measures for complexity were adapted from Shih (2008). Openness to experience was adapted from John and Srivastava (1999), while usage intention was taken from Venkatesh et al. (2012). A pretest was performed with the help of eight individuals with VR game playing experience and three experts.

## Data Analysis Results

Table 1 summarizes the means and standard deviations (SD) of the items. The construct of flow has the highest mean and lowest standard deviation for both experienced and inexperienced players, while complexity has the lowest mean. This indicates that the users in each group had similar, relatively high perceptions of flow in the VR games, and that usage of the VR device was not considered to be complex by either group. According to the independent-sample *t*-test, inexperienced players had significantly higher perceptions of flow, spatial presence, and relaxation while experienced players had significantly higher perceptions of visual fatigue and complexity.

**TABLE 1:** Statistics of the constructs.

Constructs	Inexperienced group		Experienced group		Difference
	Means	*SD*	Means	*SD*	*t*-value
Flow	4.25	0.53	3.84	0.72	5.283***
Spatial presence	3.90	0.68	3.34	0.76	6.787***
Relaxation	4.12	0.67	3.58	0.86	6.048***
Visual fatigue	2.82	0.82	3.11	0.90	−2.899***
Complexity	2.08	0.68	2.34	0.82	−2.978***
Intention	3.93	0.68	3.94	0.92	−0.157
Openness	3.86	0.53	3.93	0.67	−1.017

*** *p* < 0.001.

### The Measurement Model

In this study, we used SmartPLS 2.0 to analyze the data. Compared with traditional structural equation modeling (SEM) tools, such as AMOS and LISREL, this tool can assess the measurement and structural models simultaneously. Moreover, the partial least squares (PLS) method does not have restrict sample size and distributions. It is an appropriate approach for exploratory analysis (Chin et al., 2003). This study collected the respondents from two different groups, experienced and inexperienced VR game groups. The sample size of each group is relatively small (*n* = 150). That is the reason why the PLS approach is used in the analysis.

In the measurement model, a confirmatory factor analysis was used to examine reliability, discriminant validity, and convergent validity. With regard to the reliability, composite reliability (CR) and Cronbach’s alpha (CA) are the common criterions. The values of CR and CA ranged from 0.763 to 0.951, which exceed the 0.6 threshold for acceptable reliability (Fornell and Larcker, 1981). Analysis of the measurement model indicates the following: all items’ indicator factor loadings exceeded the accepted reliability threshold of 0.5, average variance extracted (AVE) values were within the range of acceptability (0.527–0.867), and all values for CR exceeded the accepted threshold. All the figures in the measurement model meet the conditions for convergent validity. With regard to the discriminant validity, it commonly measures the statistical difference between two factors by comparing each construct’s square root of AVE with that construct’s correlation coefficients with the remaining constructs. Proper discriminant validity requires that the correlation coefficients be less than the square root of the AVE. The analysis results show that the measurement scale fits the standard of discriminant validity.

### Structural Model and Hypothesis Testing

**FIGURE 2:**
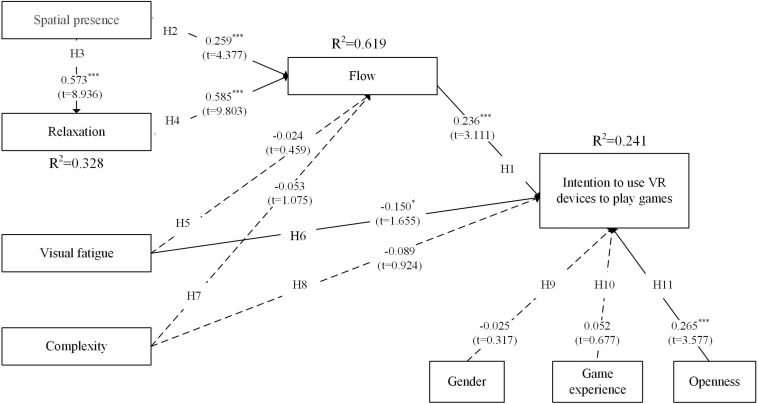
Testing results for the inexperienced player group. ^∗^*p* < 0.05, ^∗∗∗^*p* < 0.001.

**FIGURE 3:**
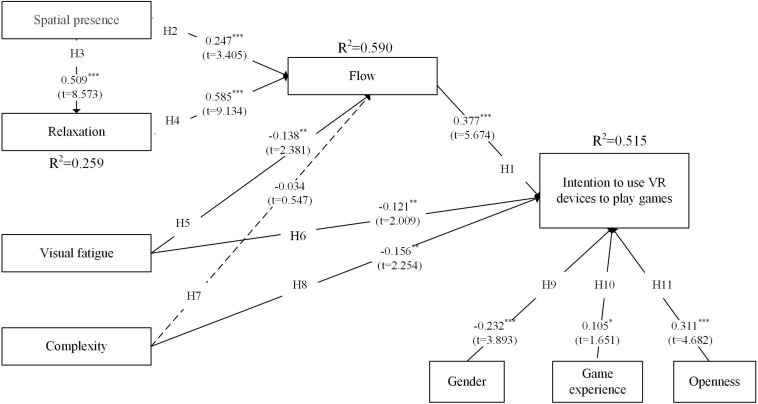
Testing results for the experienced player group. ^∗^*p* < 0.05, ^∗∗^*p* < 0.01, ^∗∗∗^*p* < 0.001.

The results for the two groups are, respectively, presented in context on the structural model in Figures 2, 3. In the structural model analysis, PLS focuses on each path coefficient and variance explained (*R*^2^) (Chin et al., 2003). In the figure, dotted lines indicate paths that are not significant. We have placed the *t*-values and path coefficients adjacent to the lines from one construct to another.

For the inexperienced player group, the benefit factor constructs of spatial presence and relaxation were found to have strong positive effects on flow, supporting H2 and H4 (β = 0.259, *p* < 0.001; β = 0.585, *p* < 0.001). Spatial presence was found to have a strong effect on both relaxation and flow, supporting H3 (β = 0.573, *p* < 0.001). In regard to the sacrifice factor constructs, however, we found that the negative effects of visual fatigue and complexity were not significant. Thus, H5 and H7 were not supported. In addition, only visual fatigue was found to have a significant negative effect on intention (β = −0.150, *p* < 0.05). Thus, H6 was supported but H8 was not. Moreover, flow was found to have a significant effect on perceived value (β = 0.236, *p* < 0.001), supporting H1. For the control variables, only openness to experience significantly affected intention (β = 0.265, *p* < 0.001). Thus, H11 was supported but H9–10 were not. Lastly, the results show that the research model explains 32.8%, 61.9%, and 24.1% of the variances of relaxation, flow, and usage intention, respectively.

For the experienced player group, the benefit factor constructs were also found to have strong positive effects on flow (β = 0.247, *p* < 0.001; β = 0.585, *p* < 0.001), thus supporting H2 and H4. Similarly, spatial presence was found to significantly affect relaxation (β = 0.509, *p* < 0.001), supporting H3. Flow also had a significant effect on intention (β = 0.377, *p* < 0.001). Hence, H1 was supported. In the constructs of sacrifice factors, visual fatigue negatively and significantly affected flow and intention (β = −0.138, *p* < 0.01; β = −0.121, *p* < 0.05). Thus, H5 and H6 were supported. Complexity had a significant direct negative effect on intention (β = −0.156, *p* < 0.05) but had an insignificant effect on flow, supporting H8 but not H7. For the control variables, we found that gender (male = 1; female = 2), game experience, and openness to experience have significant effects on intention, supporting H9–11 (β = −0.232, *p* < 0.001; β = 0.105, *p* < 0.05; β = 0.311, *p* < 0.001). Lastly, the results show that the research model explains 25.9%, 59.0%, and 51.5% of the variances of relaxation, flow, and usage intention, respectively. Of the 11 hypotheses, 6 are supported, 4 are partially supported, and 1 is unsupported.

## Discussion

This research developed a framework based perceived values to provide insight into the factors contributing to players’ intention to use VR devices to play games. The hypotheses regarding the benefit factors were supported. However, the hypotheses regarding the sacrifice factors and control variables were either partially supported or unsupported. The analysis results and findings are discussed in the following section.

### Benefit Factors

The effects of flow, relaxation, and spatial presence were strong and consistent in the models for both groups. The direct effect of flow on intention was strong and significant for both groups. This result is consistent with the findings of past mobile game studies (Hsiao and Chen, 2016; Rauschnabel et al., 2017; Fraustino et al., 2018). The influence of spatial presence on both flow and relaxation was also confirmed for both groups. The spatial presence of these VR devices can successfully enhance different players’ flow and help players to relax (Rodríguez-Ardura and Meseguer-Artola, 2016). For both groups, relaxation was found to have the strongest effect on flow. The perceptions of flow and relaxation were relatively high for both groups (>3.5). This means that users had a noticeably positive experience of flow and relaxation when using VR devices. Surprisingly, perceptions of flow, visual fatigue, and relaxation were all rated significantly higher by the inexperienced group than by the experienced group. Possible reasons for this result might include the fact that the inexperienced players were in a relatively comfortable room and that they had the help of a research assistant when using the devices. This suggests that the usage environment might affect the VR usage experience.

### Sacrifice Factors

Although the negative effect of complexity on flow has been verified in past studies, the effect was not significant for either of the two groups in this study. One possible reason might be that the perception of complexity is the lowest (<2.5) while using VR devices. The complexity may not be a significant negative factor in the young user group. Visual fatigue was also rated relatively low (<3) by the inexperienced group because the players used the devices for only about 20 min. Hence, these two perceptions were not likely to have a significant negative effect on the players’ flow state. However, the experienced group rated the perception of visual fatigue significantly higher, and visual fatigue was found to have a significant negative effect on the players’ flow. Visual fatigue was also found to have a significant negative effect on the intention of both groups. We found inconsistent results for the factor of complexity: the negative effect of complexity on the play intention was significant only for the experienced group. The perception of complexity is 2.08 and 2.34 in the inexperienced group and experienced group, respectively. Obviously, experienced users are more critical and feel that the user interface of the device/application is not simple enough. We infer that the experienced game players are likely to consider the complexity of VR devices before using them.

### Control Variables

Of the three control variables, openness to experience had the strongest effect on the intention of both groups. In general, past studies have found that the personality trait of openness influences users’ intention to adopt new technology. Many VR devices are innovative, and advanced devices such as the hTC VIVE pro are expensive. People who score low on openness tend to be conventional and are less likely to intend to adopt innovative technology (Butt and Phillips, 2008). Compared to female players in the same group, male players in the experienced group are more likely to have the intention to play VR games. In many computer games, male players are the majority. They like to test and play new and innovative games. Experienced game players also have more opportunities to play VR games. These results are consistent with the findings of past research (Hsiao and Chen, 2016). In the inexperienced group, the players have less VR game experience, and female players are the majority. Therefore, the two factors, gender and game experience, had no significant effects on the intention.

### Implications for Academic Researchers and Practice

The analysis results show that the influences of the benefit factors on the intention are consistent between the two groups. All of our hypotheses regarding the positive influences are supported. Spatial presence was found to directly affect not only users’ perception of relaxation but also their perception of flow. Spatial presence was clearly found to be more influential than relaxation in terms of VR usage behaviors. Spatial presence is the key determining factor for VR devices. Hence, the effects of spatial presence on the other benefit factors merit further study. The negative effects of visual fatigue on intention were also confirmed for both groups. Visual fatigue was found to have a significant direct effect on VR device usage intention. Visual fatigue in the VR environment might also have negative effects on the other factors or cause other problems. Future studies should confirm these negative effects. Compared with visual fatigue, complexity was found to have less of an effect on flow and intention, overall. However, the effect of complexity might differ, depending on the usage context. Since many users are not familiar with VR applications, the influence of complexity should not be ignored.

Moreover, the results show that the following factors influence the VR device usage intention of less experienced users: flow, visual fatigue, and openness to experience. In order to attract the inexperienced users to adopt VR devices, VR product developers must focus on players whose personality type is open to experience. VR products should be designed in such a way that the effects of visual fatigue are minimized. Our research model was found to have stronger explanatory power for the experienced player group: except for H7, all the hypotheses were either partially or fully supported. Our results can help VR companies develop product strategies. The results clearly show that flow is still the most influential factor in VR contexts. Relaxation was found to be a key factor of flow, and had the strongest influence. Marketers can segment their markets and design promotions based on the factors of gender, game experience, and openness.

To sum up, three suggestions about VR devices/applications are proposed according to the analysis results. First, the hardware/firmware of VR devices should be continuously upgraded. The upgraded hardware, such as higher resolution and refresh rate of the screen, can reduce the visual fatigue. Second, the user interface of the VR devices/applications should be simpler. The creation of simple user experience is crucial to user retention. Third, relaxation-related factors should be considered in the VR application development. The unique relaxation experience created by VR can enhance flow experience and play intention.

## Research Limitations

The limitations of this study are listed as below. First of all, respondents’ participation was voluntary so the sampling process is a non-random process. The analysis results may not adequately represent all players’ responses. Then, this study focused on VR games. VR devices and applications are used in many different fields, such as education and healthcare. The users’ perceptions may be different in different fields. Third, the research framework was developed based on benefit and sacrifice factors. Other factors in different theories, such as media richness theory, could be investigated and examined. Forth, our research subjects were the VR players in the central Taiwan; thus, cultural and lifestyle characteristics specific to Taiwan may have influenced the results. More investigation can be made in different countries in the future to enhance the generalizability of the findings.

## Data Availability Statement

The raw data supporting the conclusions of this article will be made available by the authors, without undue reservation, to any qualified researcher.

## Ethics Statement

The studies involving human participants were reviewed and approved by the National Taichung University of Science and Technology. The patients/participants provided their written informed consent to participate in this study.

## Author Contributions

C-CL contributed to the research topic and the methodology. K-LH contributed to the research model and the experimental design and results. C-CC contributed to the statistical analysis and the discussion.

## Conflict of Interest

The authors declare that the research was conducted in the absence of any commercial or financial relationships that could be construed as a potential conflict of interest.
